# Assessment of the Role of C3(H_2_O) in the Alternative Pathway

**DOI:** 10.3389/fimmu.2020.00530

**Published:** 2020-03-31

**Authors:** Karin Fromell, Anna Adler, Amanda Åman, Vivek Anand Manivel, Shan Huang, Claudia Dührkop, Kerstin Sandholm, Kristina N. Ekdahl, Bo Nilsson

**Affiliations:** ^1^Rudbeck Laboratory, Department of Immunology, Genetics and Pathology, Uppsala, Sweden; ^2^Department of Medical Biochemistry and Cell Biology, Institute of Biomedicine, Sahlgrenska Academy, University of Gothenburg, Gothenburg, Sweden; ^3^Linnaeus Center of Biomaterials Chemistry, Linnaeus University, Kalmar, Sweden

**Keywords:** complement, C3, C3(H_2_O), C3b, alternative pathway, C3 convertase

## Abstract

In this study we investigate the hydrolysis of C3 to C3(H_2_O) and its ability to initiate activation via the alternative pathway (AP) of the complement system. The internal thioester bond within C3 is hydrolyzed by water in plasma because of its inherent lability. This results in the formation of non-proteolytically activated C3(H_2_O) which is believed have C3b-like properties and be able to form an active initial fluid phase C3 convertase together with Factor B (FB). The generation of C3(H_2_O) occurs at a low but constant rate in blood, but the formation can be greatly accelerated by the interaction with various surfaces or nucleophilic and chaotropic agents. In order to more specifically elucidate the relevance of the C3(H_2_O) for AP activation, formation was induced in solution by repeated freeze/thawing, methylamine or KCSN treatment and named C3(x) where the x can be any of the reactive nucleophilic or chaotropic agents. Isolation and characterization of C3(x) showed that it exists in several forms with varying attributes, where some have more C3b-like properties and can be cleaved by Factor I in the presence of Factor H. However, in common for all these variants is that they are less active partners in initial formation of the AP convertase compared with the corresponding activity of C3b. These observations support the idea that formation of C3(x) in the fluid phase is not a strong initiator of the AP. It is rather likely that the AP mainly acts as an amplification mechanism of complement activation that is triggered by deposition of target-bound C3b molecules generated by other means.

## Introduction

The alternative pathway of complement (AP) is initiated by C3b and factor B forming a Mg^2+^-dependent complex as reviewed in Lachmann (1) and Harrison (2). This initial complex formation is followed by the cleavage of factor B by factor D into Ba and Bb to form the active labile enzymatic complex C3bBb, the AP C3 convertase, which in the fluid phase has a half-life of 90s as determined *in vitro* using purified components (3, 4). The C3bBb convertase can then cleave native C3 molecules into C3a and C3b. These C3b molecules trigger a positive feedback loop reaction, with each new C3b molecule potentially being able to form a new AP convertase complex.

Thus, in order for the AP to commence and form an initial AP convertase, C3b needs to be available in the fluid phase. As an indication of C3b formation, the anaphylatoxin C3a/C3a_desArg_ is constantly generated with a half-life in plasma of ~30 min (5). The C3a levels are elevated in proportion to the concentration of C3 (i.e., the C3a/C3 ratio is constant) which was evident in a normal/obese population with a wide range of C3 concentrations in the blood plasma (6). This turn-over of C3 has been explained by the tick-over theory, put forward by Lachmann et al. in the early 1970s (7, 8). This theory states that low amounts of C3b are constantly generated in sufficient quantity to be able to interact with Factor B (FB) and initiate an initial fluid-phase AP convertase. The origin and configuration of this C3 species has not been fully elucidated, but in the early 1980s Pangburn et al. described the continuous hydrolysis of the internal thioester in C3, generating a “C3b-like” molecule with no hemolytic activity (9, 10). Based on these findings, the tick-over of native C3 to C3(H_2_O) has been the prevailing mechanism explaining the tick-over theory and activation of the AP.

The C3b-like activity of C3(H_2_O) was linked to its ability to bind FB and form an AP convertase and to its susceptibility to cleavage by Factor I in the presence of Factor H. The convertase forming properties were assessed in a purified system by mixing C3(H_2_O), FB and Factor D (FD) to form AP convertases. The generated AP convertases were allowed to cleave native C3. The remaining native C3 after cleavage of C3 to C3a and C3b with C3(H_2_O)Bb was measured in a sensitive hemolytic assay (10).

One of the caveats in the earlier studies was that in all the presented experiments, the reaction was amplified by either isolated C3 nephritic factor (C3Nef) or by purified properdin, which both stabilize the AP convertase. None of these components are present under physiological conditions. C3Nef is related to C3 glomerulonephritidis (C3GN) and, as later shown, purified properdin preparations contain a large fraction of aggregates (P_n_), which cause fluid phase complement consumption when added to serum in contrast to the physiological oligomer forms (P_2_, P_3_, and P_4_) (11). More recently, the non-physiological activity of the aggregates of properdin were confirmed since P_n_ and unseparated properdin were shown to bind to numerous surfaces, in contrast to the P_2_-P_4_ forms which showed selectivity for zymosan and necrotic cells (12).

Also, in later studies it was shown that C3(H_2_O) consists of a mixture of C3 populations; one which has a native C3-like configuration C3(H_2_O^*^) and one that has a “C3b-like” form (13). The former one can return to the shape of native C3 with hemolytic activity, while the latter one is in an irreversible “C3b-like” state. This means that the preparation will contain various forms of C3 including contaminating native C3 that allows cleavage of C3 into C3b which will be able to participate in the generation of AP convertases thereby distorting an accurate evaluation of the true properties of C3(H_2_O).

**Figure 1:**
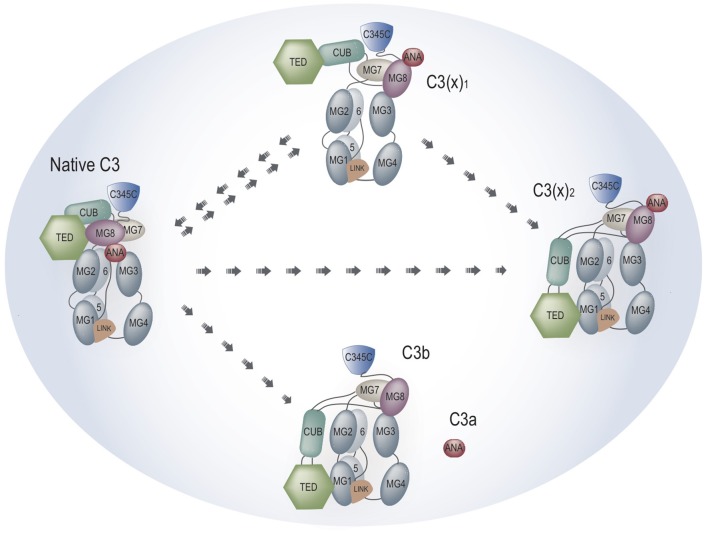
Schematic illustration of the different forms of non-proteolytically activated C3 i.e., C3(x).

In order to more precisely elucidate the relevance of C3(H_2_O) for AP activation we prepared different forms of C3(H_2_O), which from now on will be called C3(x) where the x is the reactive nucleophilic or chaotropic agent. We separated the various populations of C3(x), and demonstrated that the conditions previously described to create C3b-like molecules were variable and insufficient in order to convert all native C3 to C3(x). Confirming previous studies (13, 14), different fractions of C3(x) were identified and it was also shown that different agents [methylamine, KSCN, repeated cycles of freezing/thawing (F/T)] generated its distinctive populations. A schematic illustration of the structural rearrangement of the different forms of C3(x) is shown in Figure 1. In general, we show that all forms of C3(x) were able to form C3(x)Bb convertases and were susceptible to cleavage to Factor I in the presence of Factor H, but all forms were much more sluggish compared to C3b and no AP convertase activity was observed in the presence of Factor I and Factor H. Theoretically, only one initial C3(x) molecule is needed to start the positive feedback loop and to commence AP activation, but due to its inefficiency, this mechanism as demonstrated on various activating surfaces, is likely to be largely overruled by C3b generation mediated by the surface-bound classical pathway (CP) lectin pathway (LP) convertase. This implies that the AP mainly is an amplification mechanism (15), apart from situations when activation is dependent on insufficient regulation as in paroxysmal nocturnal hemoglobinuria (PNH), atypical hemolytic uremic syndrome (aHUS), age-related macular degeneration (AMD), etc.

## Materials and Methods

### Construction and Validation of an Assay for Detection of Non-proteolytically Activated C3 [C3(x)]

We have previously generated monoclonal antibodies (mAbs) to specific epitopes along the C3 polypeptide chains (16, 17). One of those is mAb 4SD17.3, which is specific for a neoepitope in the C3a fragment. In addition to free C3a, mAb 4SD17.3 also reacts with the C3a moiety exposed in C3 where the thioester has been disrupted, but not with intact, non-activated C3 in the fluid phase. The generated C3(x) was detected in three different ways: (1) by ELISA using monoclonal antibody (mAb) 4SD17.3 toward a neo-epitope in C3a for capture and biotinylated polyclonal (pAb) anti-C3d (Dako, Glostrup, Denmark) together with streptavidin-HRP (GE Healthcare, Uppsala, Sweden) for detection; (2) by Magpix® Luminex technology, using MagPlexC® Microspheres (Luminex, Bio-Rad Laboratories, Hercules, CA, USA) pre-coupled with mAb mouse anti-human C3a (4SD17.3) for capture (3 μg/1.25 × 10^6^ beads) and 4 μg/mL biotinylated pAb anti-C3c (Dako, Glostrup, Denmark) followed by PE-conjugated streptavidin (Bio-Rad Laboratories, USA) for detection, and (3) using the same assay but biotinylated pAb rabbit anti-human C3d (Agilent Technologies, Inc., Santa Clara, CA, USA) instead of anti-C3c.

Purified C3 (18) in which the thioester had been disrupted by repeated (10 times) freezing (−20°C) and thawing [room temperature (RT)] was dissolved in PBS (Phosphate buffered saline, pH 7.4) and used as standard in these assays. In hemolytic tests for the CP and the AP (19) this preparation of C3 was found to be devoid of activity, confirming that the thioester was disrupted.

### Assessment of the Rate of C3(x) Generation

The C3(x) ELISA was then used to measure the rate at which C3(H_2_O) is formed in plasma. Freshly drawn human whole blood in K_2_-EDTA Vacutainers® (BD, Plymouth, UK) from healthy volunteers was centrifuged to obtain plasma. The plasma was then incubated continuously rotating at 20 rpm at 37°C in PVC tubes pre-coated with heparin (Corline Systems AB, Sweden) without a bubble and samples were collected for C3(x) ELISA analysis at different time points from 0 to 180 min.

### Preparation of C3(x) With Nucleophilic and Chaotropic Agents

Native C3 [purified in-house from human plasma, according to Hammer et al. (18)] was incubated with the nucleophilic agent methylamine (0.2 M) or potassium thiocyanate (KSCN; 0.33 M) for 30 min at 37°C in VB^++^ (Veronal-buffered saline containing 5 mM Na-barbiturate, pH 7.4; 145 mM NaCl; 0.15 mM Ca^2+^; 0.5 mM Mg^2+^) adjusted to pH 8.0. After the incubation the C3 was dialyzed back to VB^++^ pH 7.3. Some of the methylamine treated C3 was subjected to repeated F/T cycles (10 times from −20°C to RT) to remove any traces of native C3.

### Size Exclusion Chromatography

The binding of the mAbs 7D84.1 (0.25 μM) or 4SD17.3 (0.25 μM) (20, 21) to C3b (0.5 μM) and F/T C3 methylamine (0.5 μM) was analyzed using Size Exclusion Chromatography (SEC). Human fibrinogen 3 (FIB 3, Enzyme research, IN, USA) was used as a positive control for complex formation. The C3b/C3 methylamine was incubated in VB^++^ for 60 min at 37°C together with the mAb, followed by SEC using ENrich™ SEC 650 10 × 300 Column coupled to an NGC^TM^ Chromatography System (Bio-Rad, USA). VB^++^ was used as elution buffer. The collected fractions were further analyzed with ELISA by incubation in wells of high-binding microtiter plates, followed by detection using either a rabbit anti-mouse IgG HRP-conjugated antibody (Dako, Glostrup, Denmark) or rabbit-anti-hu-C3c HRP-conjugated antibody (Dako, Glostrup, Denmark).

### Ion Exchange Chromatography

Cation-exchange chromatography was used to identify different C3 populations within native C3, C3 (methylamine), C3 (methylamine F/T), and C3(KSCN). The chromatography was performed using a Mono S 5/50 GL column (GE Healthcare, Bio-Sciences AB, Uppsala, Sweden) coupled to the NGC^TM^ Chromatography System. The flow-rate was set to 0.5 mL/min at RT and a gradient was established from 0 to 0.85 M NaCl in 20 mM Phosphate buffer pH 6.8. Fractions were collected for further analysis.

### Identification of C3(x) Forms in Human Serum

Human whole blood from a healthy volunteer was collected in Serum/Cat BD Vacutainers® (BD, Plymouth, UK). The collected serum was incubated at 37°C for 24 h, followed by protein precipitation using 15% polyethylene glycol (PEG)-4000 at 4°C for 60 min and then centrifuged at 13,000 × g for 10 min. Finally, the pellet was resuspended in 20 mM PBS pH 6.8. The protein solution was diluted 1:3 before adding it to the NGC^TM^ Chromatography System with the MonoS column. The same “Cation exchange MonoS C3” protocol as described above was used (Flow-rate 0.5 mL/ min, 85% gradient (0–0.85 M NaCl in 20 mM Phosphate buffer pH 6.8). The fractions were collected (25 fractions), for further analysis of C3 and C3(x). The C3c-ELISA was performed according to the protocol for detection of C3 levels described in Henningsson et al. (22). The fractions were diluted 1/200 and added to 96-well plate coated with polyclonal antibody anti-C3c (Dako, Glostrup, Denmark). This was followed by detection using biotinylated anti-C3c diluted 1/6,400 and streptavidin conjugated to HRP (GE Healthcare, Chicago, IL, US). A serum pool (consisting of 46 donors' serum) at a concentration of 500 μg/L was used as a standard. The C3(x) analysis was performed according to the assay described above.

### Wes Capillary Electrophoresis/Blotting Immunoassay

An automated Western blot-like assay was used. Simple Western 12–230 kDa size assay cartridges under reducing conditions were carried out using a Wes® analyzer (Protein Simple, Santa Clara, CA, USA) according to the manufacturer's manual. In brief, C3/C3b/C3(met)/C3(KSCN) (2.8 μg) were incubated together with Factor B (4 μg) and Factor D (0.02 μg) (Complement Technology Inc, Tyler, TX, USA) in VB^++^ at 37°C and samples were collected after 1, 5, 15, 30, 60, and 120 min. Following incubation, the samples were diluted 5x in 0.1 x Wes Sample Buffer to the final concentrations seen in Table 1. In order to follow the cleavage of FB to Bb and Ba, a mAb mouse anti-human complement factor Bb (10 μg/mL) (Bio-Rad, Kidlington, UK) detecting both intact FB and the Bb cleavage product, was used as primary antibody for the Wes analysis together with the Wes anti-mouse detection module. The following electrophoretic protein separation and immunodetection were performed using the default SimpleWestern™ settings. The quantified immune-detected signal, i.e., area under the curve, was analyzed using the Compass software (version 4.0.0, ProteinSimple™), which also converted the electropherograms into virtual blots. The experiments are repeated four times.

**Table 1:** The molecular weight, and final concentration of complement proteins used in Wes immunoassay for analysis of C3 convertase formation.

**Protein**	**Molecular weight (kDa)**	**Concentration for incubation at 37^**°**^C (μg/mL). Diluted in VB^**++**^**	**Final concentration in Wes Diluted 1:5 in 0.1 x Wes Sample Buffer (μg/mL)**
Native C3	185	70	14
C3b	176	70	14
C3 methylamine	185	70	14
C3 KSCN	185	70	14
Factor B	93	100	20
Factor D	24	0.5	0.1

### Factor I Cleavage Analysis

Cleavage of native C3, C3b, C3(met), F/T C3(met) (167 μg/mL) by Factor I (17 μg/mL, Complement Technology Inc) in the presence of Factor H (33 μg/mL, purified in-house from human serum), was analyzed by SDS-PAGE electrophoresis on a 4–20% gradient gel (Mini-PROTEAN® TXG™ Precast Gels, Hercules, CA, USA, Bio-Rad), essentially according to Hammer et al. (18). The samples were boiled under reducing conditions using 100 mM DTT, and the proteins were visualized on the gel using Coomassie brilliant blue staining.

### Hemolytic Assay of the Alternative Pathway

The hemolytic tests for the AP were performed as in Nilsson and Nilsson (19). In short, 50 μL of C3 depleted serum (Complement Technologies Inc., USA), were carefully mixed with 25 μL of C3 (positive control), C3b (negative control) or the C3(x) preparations, i.e., after treatment with methylamine and F/T. Native C3 was tested at 5 different concentrations (75–365 μg/mL final concentration) and C3b, C3(x) methylamine and C3(x) F/T were added at a final concentration that was in the middle of this concentration range (see Table 2). The serum samples were then added to 100 μL 50% rabbit erythrocytes (v/v) and agitated at 37°C for 20 min and then stopped by the addition of VB-EDTA. The activity of the test serum was compared to that of a reference serum and the activity was expressed in percent.

**Table 2:** Hemolytic assay of AP with C3 added to C3 depleted serum as indicated.

**Sample**	**Concentration (μg/mL)**	**Activity (%)**
Native C3	365	95
	295	80
	225	55
	145	51
	75	22
C3(x) methylamine	150–165	2
C3(x) F/T	230	0
C3b	145	0
Control (VBS^++^)	0	0

### Estimation of C3(x) Formation at Different pH

C3 (100 μg/mL) was incubated in Sodium Phosphate buffers with pH ranging from 4.3 to 7.3 for 60 min at 37°C. The level of C3(x) formation was measured using multiplex xMAP according to the protocol described above. The ability of these preparations to cleave FB after addition of FD was measured by Wes immunoassay. Similarly, FB consumption was also measured after first neutralizing the C3 preparations to pH 7.4. The samples were prepared for the Wes immunoassay by mixing the C3 preparations treated at pH 4.3–7.3 (70 μg/mL), FB (100 μg/mL), and FD (0.5 μg/mL) and followed by incubation at 37°C for 5, 30, and 60 min and a 5x dilution in 0.1 x Wes Sample Buffer. Wes immunoassay was performed under reducing conditions and for detection, the primary mAb mouse anti-human complement factor Bb (1 μg/mL) was used together with the Wes anti-mouse detection module.

### Generation of C3(x) in Plasma by Incubation With Nucleophilic Agents

Blood from healthy volunteers, who had not been receiving any medication for a minimum of 10 days prior to donation, was collected in Vacutainer® tubes (BD, Plymouth, UK) in the presence of the specific thrombin inhibitor lepirudin (50 μg/mL, Refludan^TM^, Aventis Pharma) and centrifuged to obtain plasma. Aliquots (20 μL) of physiological relevant final concentrations of ammonium hydroxide solution (0–3.2 mM) were added to 480 μL of lepirudin plasma and incubated for 60 min at 37°C. The levels of generated C3(x), C3a and sC5b-9 in the samples were analyzed by ELISA. C3a and sC5b-9 were assessed according to Nilsson et al. (21) and Mollnes et al. (23). Each experiment was performed separately using blood from different donors.

### Activation of the Alternative Pathway on Different Surfaces

Four different surfaces, i.e., lipopolysaccharide (LPS) coated polystyrene (PS), bare PS, glass, and polypropylene (PP) tubes were selected for the evaluation of surface-induced activation of the AP in lepirudin plasma. The LPS coated tubes were prepared by adsorption of LPS (1 mg/mL) from *Escherichia coli* O55:B5 (Sigma Aldrich) to clean PS tubes during 1h at RT, followed by careful washing with PBS. All tubes were dried before use. The lepirudin plasma was added to the four different types of tubes and incubated stationary at 37°C. Two parallel series of experiments were performed; one with the addition of Mg-EGTA (0.5 and 10 mM final concentration, respectively) and the other without, where the volume was compensated with the corresponding amount of PBS. Samples were collected after 0, 15, 30, 60, and 120 min. EDTA (10 mM final concentration) was immediately added to stop further activation. The surface mediated complement activation was monitored as the generation of C3a measured by sandwich ELISA. The experiment was repeated with three different blood donors.

### Statistics

All experiments have been repeated at least 3 times. Data are presented as mean values ± SEM or as a representative image. Statistical calculations (one-way ANOVA with Bonferroni's multiple comparisons test) were made using GraphPad Prism version 6.0 (GraphPad Software, La Jolla, CA USA). *P* < 0.05 was considered significant. Correlation between the different parameters was calculated with the non-parametric Spearman correlation test. Differences between groups (patients vs. controls) were calculated using the Mann-Whitney U-test.

### Ethics

Ethical approval for blood collection was obtained from the regional ethics committee in Uppsala with the diary number 2008/264.

## Results

### Validation of an Assay for Non-proteolytically Activated C3 [C3(x)]

**Figure 2:**
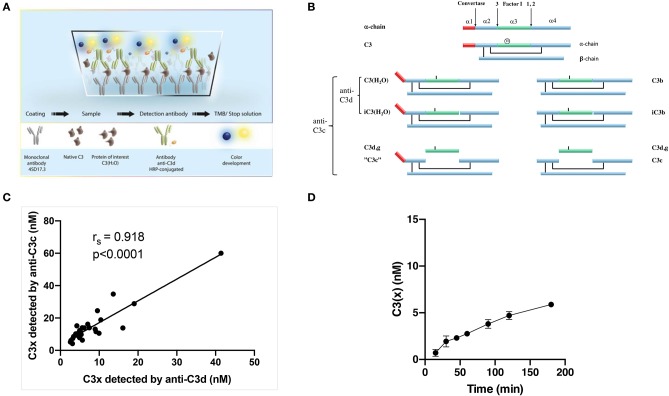
**(A)** An illustration of the newly developed assay to measure non-proteolytically activated C3. **(B)** Schematic linear representation of native C3 [α-chain alone with indicated cleavage sites for convertases and Factor I appointed 1–3 (top of the figure) and the intact C3 molecule with a mark for the position of the thioester (below)], non-proteolytically activated C3 i.e., C3(x) (left) and C3 activated by convertases i.e., C3b (right) and their degradation fragments. The figure is adapted from Ekdahl et al. (25) with permission from the publisher. **(C)** Correlation between measured levels of C3(x) using anti-C3d or anti-C3c polyclonal antibodies for detection. **(D)** Measured C3(x) formation in plasma over time from 0 to 180 min.

An ELISA for detection of C3(x) (24) was used employing the monoclonal mAb 4SD17.3 against a neo-epitope in C3a for capture and polyclonal antibody anti-C3d for detection (Figures 2A,B). Since the epitope for mAb 4SD17.3 is exposed both in high molecular weight C3(x) and low molecular weight C3a, a mix-up between C3(x) and C3a is possible. Although this interaction occurs only to a small extent, an initial PEG precipitation step was included prior to analysis. In this study, the assay was transferred to the Mag-pix platform using the same pair of antibodies. Good correlation was found between the results obtained with the two techniques (r_s_ = 0.976, *p* < 0.0001), albeit with higher nominal values found for the ELISA. When fully optimized, the intra-CV for the assay was 2.7% and the inter-CV 6.0%. Next, we tested whether the choice of detection antibody (anti-C3d or anti-C3c depicted in Figure 2B) would affect the levels of detection. The rationale is that bound C3b and C3(x) are susceptible to cleavage by Factor I in plasma which ultimately may cleave off C3c. This can potentially result in lower nominal values if anti-C3d is used for detection. As this was not the case, the values correlated closely (r_s_ = 0.918, *p* < 0.0001) with only slightly higher values obtained when anti-C3c was used for detection (Figure 2C).

The ELISA was then used to monitor the spontaneous formation of C3(x) in plasma. The formation of C3(x) is easily affected by external factors such as the activating surfaces e.g., those presented by the walls of the reaction tubes or at the air-liquid interface. We therefore carefully selected low-adsorbing polypropylene tubing and removed all air bubbles before onset of the experiment to ensure that it actually was the spontaneous formation of C3(x) that was analyzed with minimal influence from other factors. This may explain the slower rate of C3(x) formation obtained in our experiments compared to values previously published (10). There was an initial faster C3(x) generation which tended to reach a slower, stationary stage within 60 min. Overall it showed a slow but continuous C3(x) formation of ~3 nM per hour (Figure 2D).

### Characterization of C3(x) Preparations

Nucleophilic and chaotropic agents have been proven to accelerate C3 conversion to C3(x) in selected composition buffers (9, 10). C3(x) was prepared by treatment with either the chaotropic agent KSCN [C3(KSCN)] or the nucleophilic agent methylamine [C3(met)]. In addition, C3(x) prepared by repeated F/T and C3(met) also exposed to repeated F/T were included in the study.

#### Size Exclusion Chromatography

**Figure 3:**
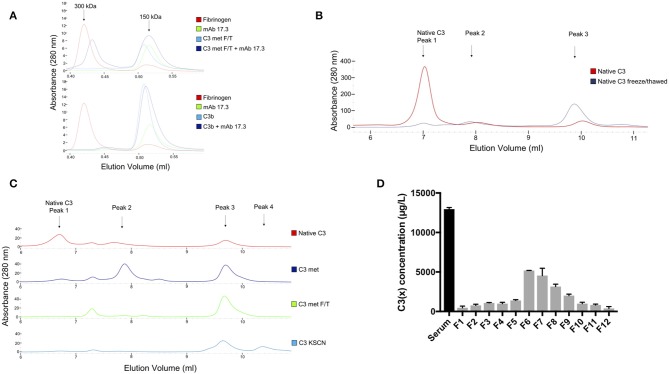
**(A)** SEC analysis of complexes formed between anti-C3a mAb 4SD17.3 and F/T C3 (upper panel) and C3b (lower panel). Fibrinogen with a molecular weight of ca 300 kDa was used as a molecular weight marker with approximately the same size as formed complexes. **(B)** MonoS cation exchange chromatography analysis of native C3 (red line) and C3 after repeated F/T (blue line). **(C)** The corresponding MonoS analysis of native C3 (red line), methylamine treated C3 (darkblue line), methylamine treated C3 after repeated F/T (green line) and KSCN treated C3 (lightblue line). The identified main peaks are marked in the chromatogram. **(D)** C3(x) ELISA for identification of C3(x) in the fractions containing C3 collected from MonoS cation exchange chromatography separation of human serum sample after incubation at 37°Cover night (24 h). The serum sample was precipitated with PEG 4000 prior to application on the column.

In order to characterize the C3(x) preparations, C3(x) prepared by methylamine treatment and repeated F/T were allowed to bind to mAb 4SD17.3. This antibody is directed toward a neo-epitope of C3a only exposed on C3(x), but not in native C3. The proteins before and after complex formation were analyzed on a SEC column. Fibrinogen (molecular weight of 300 kDa) was used as a molecular weight marker as it eluted at approximately the same potential volume as the formed complexes. As seen in the Figure 3A (upper panel), C3(x) with a molecular weight of 180 kDa is eluted slightly before the antibody (150 kDa). However, after incubating C3(x) with mAb 4SD17.3, complexes were formed which appeared as a distinct peak near the 300 kDa molecular weight marker proving that the C3a domain was exposed and available for binding. It was confirmed that the 300 kDa peak contained complexes between C3(x) and mAb 4SD17.3, by detection of both C3 and IgG in the fractions collected from the SEC analysis using ELISAs (Supplementary Figures 1B–E). C3b, lacking the C3a fragment, was used as a negative control (Figure 3A, lower panel) and as expected, no complexes were formed after incubation with the mAb 4SD17.3. Similarly, complexes were allowed to form between C3(x)/C3b and mAb 7D84.1, which is directed against a neo-epitope in C3d,g, only available in the denatured form of C3. Separation of the SEC column shows no traces of complexes at the 300 kDa elution point, indicated by fibrinogen (red trace). Instead, C3 (blue trace), mAb 7D84.1 (green trace) and the mixture of the two after incubation (dark blue trace), were all eluted at the same position. The same pattern was seen for the corresponding analysis of C3b and mAb 7D84.1 (Supplementary Figure 1A). These results show that the C3a epitope is available in the C3(met) preparation, but the molecule has not undergone a conformational change that exposes the neo-epitope in C3d,g.

#### Ion Exchange Chromatography

The elution profile of native C3 (Figure 3B, red line) separated on a MonoS cation exchange chromatography column shows one distinct peak (peak 1) that contains native C3 followed by two small peaks (peak 2 and 3) that contain C3(x) since a small part of the C3 population usually undergoes spontaneous hydrolysis. In the corresponding analysis of a sample where C3(x) had been formed by repeated F/T (Figure 3B, blue line), the vast majority of the sample population was instead eluted in peak 3, a small part still appeared in the second peak, while only a minor part remained in the first peak. Separation profiles of the different C3(x) preparations, i.e., C3(met), F/T C3(met), and C3(KSCN) on the MonoS column are shown in Figure 3C. The methylamine treated C3 was mainly eluted in peak 2 and peak 3, but repeated F/T of this sample caused most of the population in peak 2 to be transferred into peak 3. In addition, there is a small peak between peak 1 and 2. This peak increases slightly after freezing/thawing cycles, while peak 1 that contains native C3 disappears (Figure 3C, green line). Although this peak has not been characterized due to the small amount, it is assumed to contain C3(x) or a derivative thereof. A similar pattern was detected for KSCN treated C3, with the difference that this preparation also contained a 4th peak at the end of the elution profile. However, further analysis showed that this peak contained C3(KSCN) with similar functional properties as those in peak 3 in the same preparation (Supplementary Figure 3C). Since this analysis of C3(x) indicates that there are two different populations C3(x) formed initially, separated as peak 2 and peak 3 on the MonoS column, these two fractions were collected for further characterization and will in the following text be named C3(x)_1_ and C3(x)_2_ respectively.

### Identification of C3(x) Forms in Human Serum

In addition to studying C3(x) formation in a purified system, C3(x) generation in human serum was also analyzed after incubation at 37°C overnight followed by PEG 4000 precipitation before separation on the cation exchange (MonoS) chromatography column (Supplementary Figure 2A). All collected fractions were analyzed for the total amount of C3 using a C3c ELISA. The complete analysis of all the fractions from the MonoS separation is found in Supplementary Figure 2B. As illustrated in this figure, most of the C3 was eluted in fraction 2–9. In order to identify the amount of C3(x) in these fractions the C3(x) assay was used. It turned out that C3(x) was mainly found in fraction 6–8 and to some extent also in fraction 9, but only traces in later fractions (Figure 3D). This indicates that mainly the first form of C3(x) i.e., C3(x)_1_ is present in the serum sample and only minute amounts, if any, of C3(x)_2_. One reason for this may be that, in contrast to C3(x)_1_, C3(x)_2_ is easily cleaved by Factor I in the presence of Factor H and the split products will be both PEG precipitated and separated differently on MonoS columns compared to the intact C3(x)_2_ molecules.

### Generation of Bb in Presence of Different C3(x) Preparations

**Figure 4:**
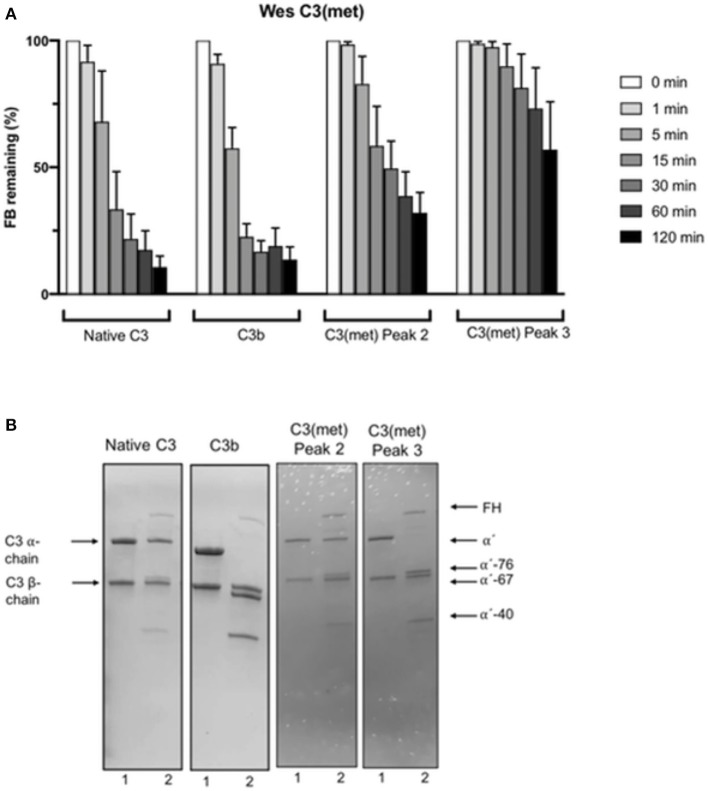
**(A)** Wes immunoassay monitoring the consumption of FB due to cleavage to Bb and Ba after the addition of FD to native C3, C3b and peak 2 and 3 isolated after separation of methylamine treated C3 on a MonoS chromatography column at different time points from 1 to 120 min. The decrease in FB due to cleavage to Bb and Ba was measured by Wes immunoassay using a specific mAb for Bb. The data is presented as mean ± SEM. **(B)** Cleavage by Factor I in the presence of Factor H of native C3, C3b and peak 2 and 3 isolated after separation of methylamine treated C3 on a MonoS chromatography analyzed by SDS-PAGE under reducing conditions. The samples before incubation are marked with “1” and the same samples after incubation with Factor I and Factor H are marked with “2” below each lane in the panel.

To get an idea of the properties of the two forms of C3(x) [i.e., C3(x)_1_ and C3(x)_2_] identified by the cation exchange (MonoS) chromatography separation, their ability to bind FB allowing its subsequent cleavage by FD into Bb and Ba and thereby forming a C3 convertase, was evaluated using a new method, similar to that employed by Pangburn previously (9), but which was modified in the detection stage. In our method, the cleavage of FB and the ensuing generation of Bb was measured with Wes immunoassay after 1, 5, 15, 30, 60, and 120 min. For comparison, native C3 and C3b were also included in the study. The results are summarized in Figure 4A. As expected, C3b readily binds FB which in the presence of FD is cleaved to Bb and Ba. Already after 1 min the amount of FB was reduced to 90%, after 5 min to ca. 60%, after 15 min only 25% remained and after 30 min almost all FB was consumed. The same scenario was seen in the native C3 sample, which can be explained by C3b contamination in the C3 preparation. Immediately after addition of FB and FD a C3 convertase forms that cleaves C3 and generates more C3b, leading to an efficient positive feedback cleavage of FB, demonstrating the accelerating nature of the AP. However, much slower FB cleavage profiles were observed with both C3(x)_1_ and C3(x)_2_ from the methylamine treated C3 samples separated on the MonoS column. In C3(x)_1_, practically all FB remains after 1 min, 85% after 5 min, 60% after 15 min and there are still 30–40% FB in the sample after 60–120 min. An even slower FB reduction was obtained in the sample with C3(x)_2_. In this case, all FB remains after 1–5 min, 90% after 15 min, and as much as 60% still has not been consumed after 120 min. It should be noted that the ability of the different C3, C3b, and C3(x) preparations to bind and cleave FB is easily affected by different factors that change the conformation of the C3 preparations. This is particularly true if the preparations are stored (e.g., at +4°C or frozen/thawed), which is illustrated in Supplementary Figure 3A. It presents the corresponding blot views of Wes immunoassays in four serially repeated experiments performed over a week. The FB cleavage was increasingly slower for all C3 preparations, but the relative difference between native C3/C3b and C3(x)_1_/ C3(x)_2_ was similar in all cases, where C3(x)_2_ was by far the least effective.

KSCN treatment of C3 did not result in any C3(x)_1_, but a large population of C3(x)_2_, which was collected from the MonoS chromatography separation and tested for its capacity to bind FB, which subsequently may be cleaved to Bb and Ba in the presence of FD (Supplementary Figure 3B). As expected, a very slow FB consumption was obtained with C3(x)_2_ from C3(KSCN), almost identical to that observed with C3(x)_2_ from C3(met), which confirms that this population of C3(x) is a poor initiator of the AP.

### Susceptibility of C3(x) to Factor I and Factor H Cleavage

To further evaluate the specific properties of C3(x), the inactivation by Factor I in the presence of Factor H was analyzed by SDS-PAGE electrophoresis under reducing conditions and stained with Coomassie Brilliant Blue. Figure 4B shows the results from Factor I + Factor H cleavage of native C3, C3b, and C3(x)_1_ and C3(x)_2_ collected as peak 2 and 3 on the MonoS column. Native C3 was used as a control, since it should not be cleaved by Factor I in the presence of Factor H, but despite this, a weak 40 kDa band was visible after incubation. This indicated that the native C3 preparation contains a small amount of C3(x), which was clearly visible in the MonoS chromatogram. Here, in addition to the first large peak with native C3, there were also several succeeding small peaks containing C3(x) (Figure 3B). An almost complete cleavage of the α-chain to a 76 kDa and a 40 kDa fragment was seen in the C3(x)_2_ sample as well as with C3b (67 and 40 kDa). On the other hand, C3(x)_1_ was only partly cleaved, since some intact α-chains remained after the incubation with Factor I and Factor H, although a weak 40 kDa band also started to appear. The same was observed in the methylamine treated C3 sample before F/T, whereas C3(KSCN) and C3(met) preparations that had been exposed to repeated F/T showed a complete cleavage of the α-chain to a 76 kDa and a 40 kDa fragment after incubation with Factor I and Factor H (Supplementary Figure 3C). Moreover, the addition of Factor I and Factor H to native C3 and C3b followed by addition of FB and FD completely inhibited the conversion of FB to Bb (Supplementary Figure 3D).

### Hemolytic Activity

In the AP hemolytic assay, native C3 added at fixed concentrations ranging from 75 to 365 μg/mL to C3 depleted serum, showed a linear relationship between the increasing C3 concentration and an upturn in activity, where the highest added C3 concentration reached 95% activity. But the C3(x) prepared either by treatment with methylamine [C3(met)] or by repeated F/T [C3(x)] was found to be devoid of activity, confirming that the thioester bond had been disrupted. The same was found for C3b. The results from the hemolytic assay are summarized in Table 2.

### C3(x) Formation as a Function of pH

**Figure 5:**
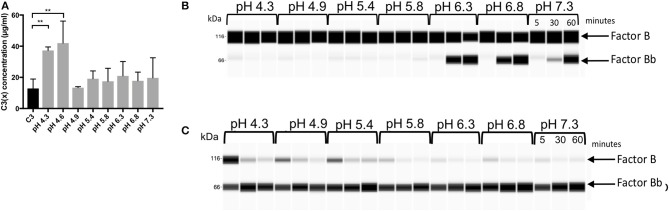
**(A)** Measurement of C3(x) formation at different pH using the C3(x) ELISA. **(B)** Virtual blot view of Wes immunoassay analysis of Bb generation after addition of FD to C3 at different pH. **(C)** The corresponding virtual blots for Bb generation after addition of FD to C3 incubated at different pH, followed by re-neutralization back to neutral pH (7.4).

To evaluate the sensitivity of the C3 thioester integrity to changes in pH, the generation of C3(x) was measured after incubating C3 in phosphate buffers with pH ranging from 4.3 to 7.3, which were selected to cover a range from an acidic intracellular milieu (e.g., lysosomes) to the neutral pH of blood plasma. The lowest pH (4.3 and 4.6) induced significant levels of C3(x) formation, while the pH of 4.9 and above did not seem to affect the C3 conformation to any measurable extent (Figure 5A). Also, the ability of these C3 preparations to form a C3 convertase and cleave FB to Bb after addition of FD within the same pH intervals was evaluated using Wes immunoassay (Figure 5B). As described above, a few molecules of C3b in the native C3 preparation are sufficient to start the convertase formation, which rapidly leads to the formation of more C3b by cleavage of C3 and a fast-continued convertase formation, measured as the conversion of FB to Bb. If there is a large proportion of C3(x) in the sample, it cannot be cleaved by the convertase and generate more C3b, which results in a slower conversion of FB to Bb. Interestingly, the most efficient generation of Bb was found at a pH between 6.3 and 6.8. The slightly higher pH (7.3) still generated Bb, but at a slower rate, whereas a pH of 5.8 and below seemed to prevent Bb formation. The lack of activity at low pH may be due to both a large formed proportion of C3(x) or that the convertase activity and complex formation is not functioning in that environment. In addition, low pH may affect the enzymatic properties of the serine proteases Bb and FD, since their active sites are likely exhibit pH sensitivity. Therefore, the same experiment was performed after first neutralizing the C3 preparations, and thereby only the correlation between C3(x) formation and the efficiency of FB cleavage/Bb generation was studied (Figure 5C). After incubation at the lowest pH (4.3) more C3 was transformed to C3(x), which resulted in slower FB cleavage/Bb generation. When the pH increased, less C3(x) was formed and the rate of FB reduction became faster. No measurable difference was seen in the C3 samples after treatment in pH 5.8, 6.3, 6.8, and 7.3, and in these cases most of the FB had been converted to Bb after 5 min.

### Generation of C3(x) in Plasma During Incubation With Nucleophilic Agents

**Figure 6:**
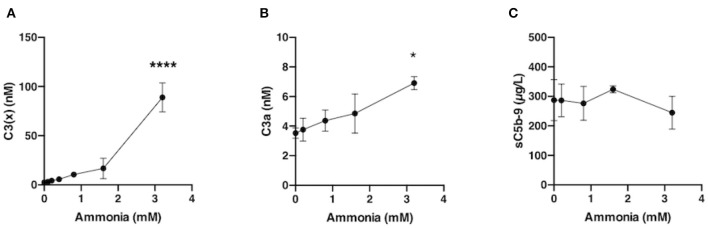
Generation of C3(x) and complement activation induced by ammonia in human lepirudin plasma. Serial amount of ammonia (0–3.2 mM) was incubated together with human plasma, and thereafter, C3(x) **(A)**, C3a **(B)**, and sC5b-9 **(C)** levels were examined. Data was collected from 3 donors and presented as mean ± SEM. Significant difference compared to control plasma without addition are indicated as *P < 0.05 and ****P < 0.0001.

As our initial experiment pointed to C3(x) in solution being a poor initiator of AP in a pure system, we decided to further investigate its functionality in plasma. This was tested by the addition of physiologically relevant amounts of ammonia (0–3.2 mM) in our model with human lepirudin plasma followed by immediate measurement of C3(x) generation. The C3(x) generation was induced by the presence of ammonia in a dose dependent manner, and a distinct increase of C3(x) was already detected at 0.2 mM ammonia (Figure 6A), which was a clinically relevant level that can be found in acute liver failure patients (26). However, a small increase in the level of complement activation marker C3a was induced (*p* = 0.0324) by the highest amount of ammonia (3.2 mM) (Figure 6B) while no effect was detected by measuring sC5b-9 (Figure 6C), suggesting that C3(x) formed by ammonia is a poor trigger of complement activation.

### Surface Induced Complement Activation in Plasma

**Figure 7:**
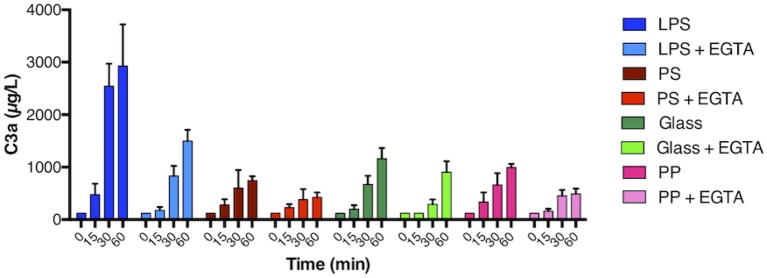
C3a generation after incubation (0–60 min) of human lepirudin plasma with different types of surfaces, with and without the addition of Mg^2+^-EGTA.

Initiation of complement activation in human plasma after contact with a number of different types of surfaces was studied by measuring the generation of C3a at different time points from 0 to 60 min. In order to evaluate how much AP contributes to this activation, the C3a levels in lepirudin plasma after surface contact were compared with corresponding plasma samples where CP and LP had been turned off by the addition of EGTA. As seen in Figure 7, the C3a levels generated after incubation with solid surfaces were generally higher at all time points in the samples where activation was allowed via CP and LP, compared to those where activation was via AP (+ EGTA). As expected, highest C3a levels were elicited by the LPS surface, a well-known complement trigger. Even though the values were significantly elevated in the EGTA-plasma, they were twice as high in plasma without EGTA. The other three tested solid surfaces, i.e., glass, PS and PP, resulted in an increase in C3a generation over time, and the measured concentrations were slightly lower when activation occurred via AP in all cases.

## Discussion

In the present study, we investigate C3(x) both in purified systems and in serum and plasma and we confirm that C3(x) exists in several forms (13). The AP convertase-forming properties and the sensitivity to inactivation by Factor I of C3(x) is much more sluggish and varies between all of these forms compared with the corresponding activity of C3b both in purified systems and in plasma. These observations support the idea that formation of C3(x) in the fluid phase is not the main mechanism by which C3b is made available during AP activation (2, 25).

A crucial question at issue was how “C3b-like” C3(H_2_O) [i.e., C3(x)] is. A caveat in the early studies is that in all the presented experiments, the reaction was amplified either by C3 nephritic factor (C3Nef), properdin or by using Ni^2+^ instead of Mg^2+^ (3). All these actions taken were natural in order to be able to characterize the C3(x)Bb convertase but make the assessment of the C3b-like activity difficult. The conclusion from these early studies is that C3(x) obtains “C3b-like” properties and that this molecule has similar activity as C3b (3, 9, 10, 27, 28), giving the impression that it is a sufficient mechanism for generating C3b and initiating AP activation. There is clear evidence that C3(x) forms a complex with FB but except for the original observations using C3Nef or purified properdin, only few examples exist that it alone actually forms an efficient active convertase (3, 9, 10, 27, 28).

In the initial studies, the rate at which the thioester was hydrolyzed was estimated to be 0.2–0.4% per hour (10) which has been confirmed in later studies including in this paper using specific ELISAs (24, 29). However, we found a lower rate of C3(x) formation in plasma (ca 3 nM/h), most likely by minimizing the influence of interfering surfaces such as the walls of the reaction tubes and the air-liquid interface. In the present study we used two western blot-like assays: first capillary electrophoresis in order to test C3(x)'s ability to bind FB, which subsequently can be cleaved by FD into Bb and Ba, and thereby form an AP convertase and secondly SDS-PAGE in order to test its inactivation by Factor I in the presence of Factor H. We found that C3b and native C3 (being converted to C3b by the formed AP convertases) both were able to almost completely convert factor B to Bb within 1 min in a fluid-phase assay. By contrast, using fully converted C3(x) i.e., no hemolytic activity, only sluggish convertase activity (without the presence of C3Nef or properdin) and Factor I inactivation was obtained with methylamine, F/T and KSCN-treated C3 compared with C3b.

Another issue in the previous literature is that “C3(x)” was generated by incubating the native C3 in the presence of 0.1 M methylamine, pH 7.4 at 37°C for 60 min, which may be a too mild condition for full conversion to C3(x) (9, 10). Supporting the hypothesis that native C3 may be remaining in the mixture is that the experiments showed a large proportion of remaining C3 α-chain after cleavage with Factor I in the presence of Factor H, given that cleavage of the α-chain by Factor I is a way to differentiate native C3 from C3(x). In the present paper, we confirm that C3 treated with methylamine according to older protocols only leads to partial cleavage in the α-chain by Factor I. Similarly, KSCN treatment of native C3 generates a similar preparation. This implicates that previous protocols do not allow full conversion of native C3 to C3(x), although this was contradicted by the low hemolytic function of the preparation.

This issue was clarified in later studies by Pangburn et al. (13) where they show that C3(NH_3_) preparations contain two major populations of C3(x); one which is able to return back to native C3 [C3(x)_1_], and the other that remains irreversibly in a C3(x) state [C3(x)_2_]. These forms have been demonstrated to have the anaphylatoxin domain (ANA) at different positions (14). The first one has properties that makes it elute at close to the same position as the native C3 position in the cation exchange (MonoS) chromatogram while the latter one, with the ANA sliding through a gap formed by the macroglobulin domains, is eluted much later (13). In our investigation we find similar populations in all C3(x) preparations in addition to smaller widely distributed populations and to native C3.

The fact that C3(x)_1_ can be converted back into native C3 makes it possible that C3b can be present or formed in the C3(x) preparations and thereby could link the C3b-like property to C3b instead of C3(x). In order to clarify if this was the case we made a similar separation on MonoS and tested the activity of the C3(x)_1_ and C3(x)_2_ population derived from the methylamine treated C3. It revealed that C3(x)_1_ had AP convertase forming capacity although to a lower extent than C3b, while it was fairly resistant to cleavage by I and H. C3(x)_2_ had even slower AP convertase activity but was much easier to cleave by Factor I and H. These experiments imply that C3(x) has C3b-like activity but is less active. This was confirmed in serum during forced C3(x) formation by adding ammonia, where only a small amount of C3a was generated. These results indicate that fluid-phase C3(x) is not a very efficient partner in the AP convertase and therefore may be a slow contributor of C3b activity.

AP activation is a surface-oriented reaction and the tick-over of C3 to C3(x) in the fluid phase would theoretically only be able to provide minute amounts of C3b to the surface, since the deposition is highly dependent on the distance during which nascent C3b is active for covalent binding. However, in order to avoid a long lag phase before the activation takes off, a large amount of initial C3b molecules bound to the target surface is needed to speed up the activation. Without available C3b molecules, the lag phase would be very extended since the C3b generation is exponentially starting from one potential molecule (30). This is very well illustrated on a biomaterial surface both *in vitro* and *in vivo* where this lag phase may last up to 5–10 min (31–33).

It is also important to take the target surface into consideration. The surface needs to allow AP activation, a function that is regulated by Factor H, Factor H-related (FHR) proteins, properdin etc. If it does not, there will be no AP activation (34). But if these molecules bind to the surface in a favorable proportion, disrupting the homeostasis between activation and regulation, AP activation may be triggered, which is the case in PNH, aHUS, AMD etc. Here a long lag phase does not have any importance for the pathology.

CP and LP activation is part of the specific attack initiated by antibodies, pentraxins, collectins, and ficolins. They are able to provide the necessary C3b molecules, which allows AP activation to start immediately as part of our defense against foreign substances such as bacteria, viruses etc. They also provide specificity to the AP which is governed by the specificity of the CP and the LP and the properties of the target surface. This also explains that the vast majority of C3b molecules, particularly in inflammatory reactions, are generated by the AP convertases, even if complement activation is initiated by the CP or LP. This indicates that the AP is mainly an amplification loop which is the essence of the AP and to a lesser extent an activation pathway *per se* (15). This notion is illustrated in Figure 7 and has recently been discussed in Ekdahl et al. (25).

## Data Availability Statement

The datasets generated for this study are available on request to the corresponding author.

## Ethics Statement

The studies involving human participants were reviewed and approved by the Regional Ethics Board in Uppsala with the diary number 2008/264. Written informed consent for participation was not required for this study in accordance with the national legislation and the institutional requirements.

## Author Contributions

KF, KE, and BN designed the research project. AA, AÅ, VM, SH, and KS performed the experiments. KF, AA, KE, and BN wrote the manuscript with editorial assistance from AÅ, VM, and CD. CD drew the graphical illustrations. All authors participated in editing the final manuscript and have read and approved the final manuscript.

### Conflict of Interest

The authors declare that the research was conducted in the absence of any commercial or financial relationships that could be construed as a potential conflict of interest.
