# Supplementary material for: Assessment of the Role of C3(H2O) in the Alternative Pathway
Source: Front Immunol. 2020 Mar 31;11:530. doi: 10.3389/fimmu.2020.00530 (PMC7136553; doi:10.3389/fimmu.2020.00530)
Supplement: Supplementary file 1 [file Data_Sheet_1.PDF]

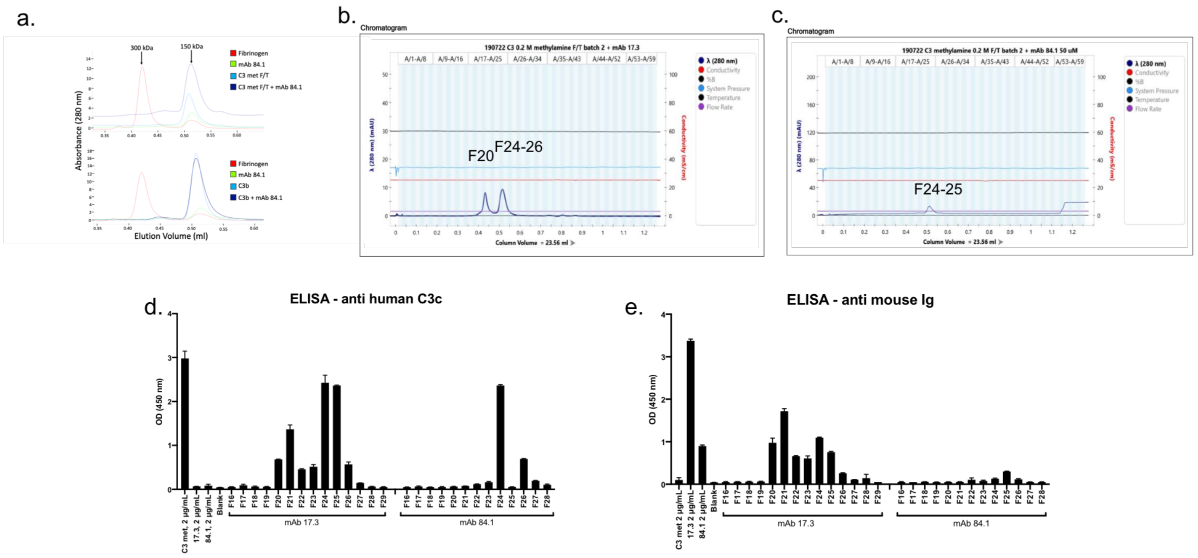

Supplement figure 1: (a) SEC analysis and separation of complexes formed between C3b and antiC3a mAb 4SD17.3 (upper panel) and neoepitope mAb 7D84.1 (lower panel). Fractions collected during the SEC analysis of complexes formed between (b) C3(met) and antiC3a mAb 4SD17.3 and (c) C3(met) and mAb 7D84.1, which then were analyzed by ELISA to determine the presence of C3 (d) and mAbs (e) in the collected fractions shown in (b) and (c).

a.

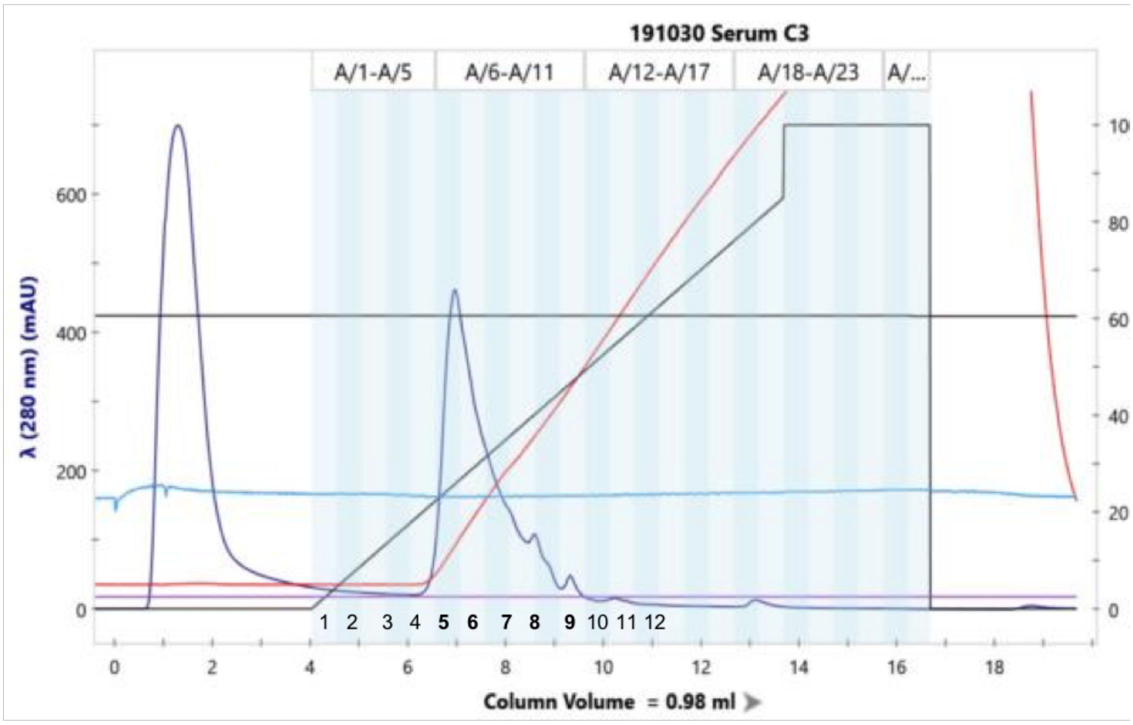

b
.

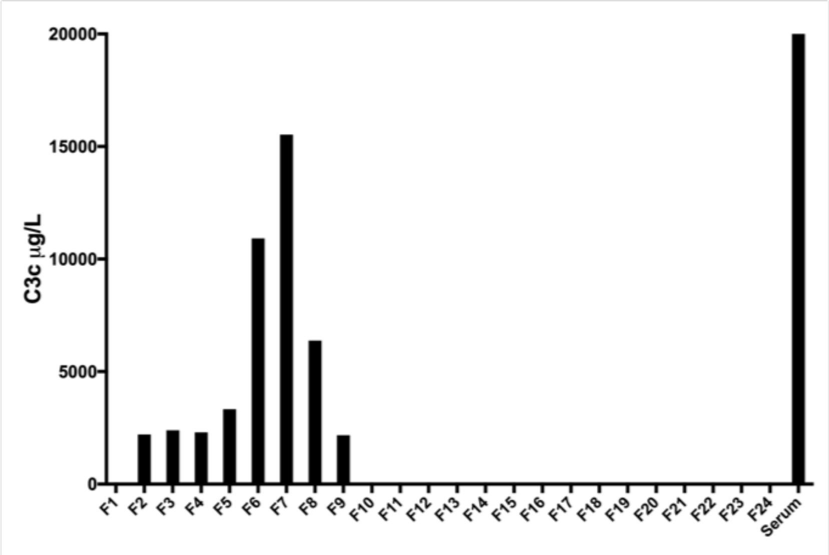

Supplement Figure 2. (a) MonoS cation exchange chromatography separation of human serum after incubation at 37°C over night and subsequent PEG 4000 precipitation. Fractions were collected throughout the elution procedure and (b) analyzed for C3 using an anti-C3c ELISA. The first 12 fractions (marked in the chromatogram) were found to contain C3 and were therefore also analyzed for C3(x) content.

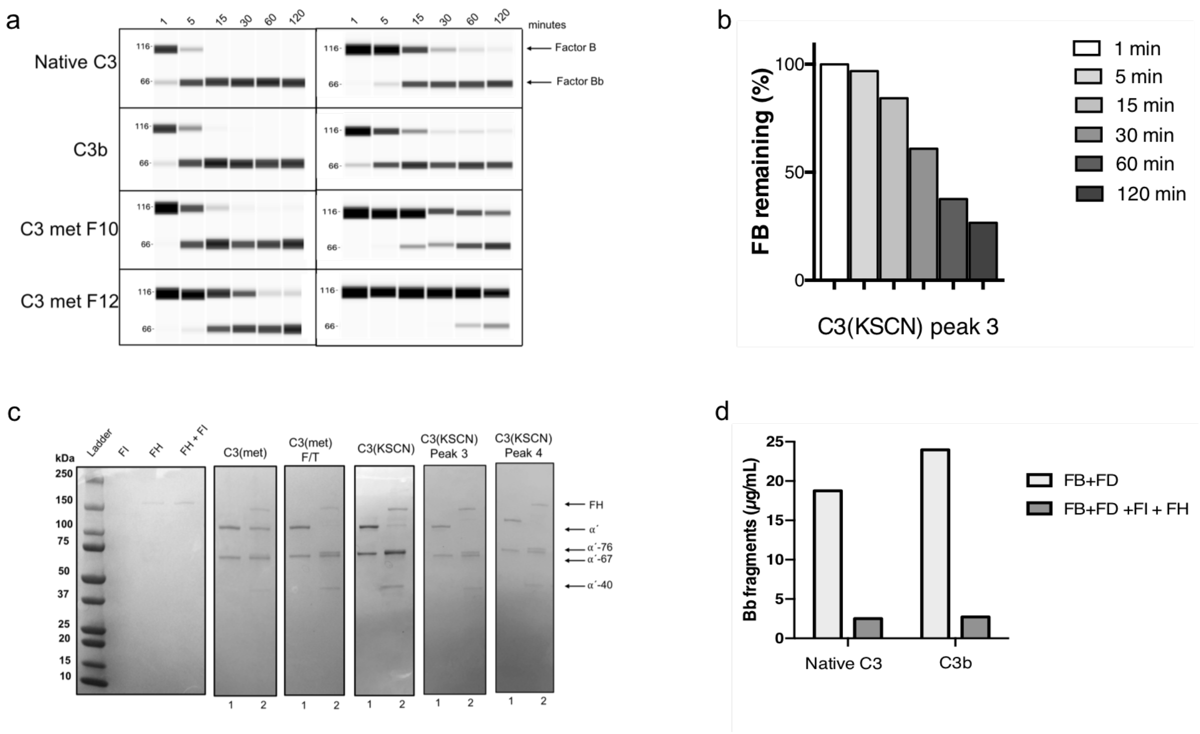

Supplement Figure 3. (a) Virtual blot view of the Wes immunoassay presented in Figure 4A, showing the fastest (panel to the left) and slowest (panel to the right) FB cleavages by C3, C3b and C3(x) peak 2 and 3 out of four analyses performed (b) Representative Wes immunoassay measuring the cleavage of FB to Bb and Ba after addition of FD to KSCN treated C3, peak 3 separated on the MonoS chromatography column. (c) Coomassie-stained SDS-PAGE electrophoresis from the left: ladder, and three references i.e. FI, FH, and a mixture of FI + FH. Then follows: C3(met) (whole sample before MonoS fractionation), freeze/thawed C3(met), C3(KSCN) (whole sample before MonoS fractionation) and C3(KSCN) peak 3 and 4 after MonoS fractionation. The lines marked with “1” are the samples before incubation and the lines marked with “2” are the same samples after incubation with FI and FH. (c) Wes immunoassay analysis of Bb fragments generated after 60 min incubation with C3 or C3b, with and without the addition of FI and FH.
